# Supplementary material for: Gut Microbiota Modulation of Moderate Undernutrition in Infants through Gummy Lactobacillus plantarum Dad-13 Consumption: A Randomized Double-Blind Controlled Trial
Source: Nutrients. 2022 Mar 1;14(5):1049. doi: 10.3390/nu14051049 (PMC8912314; doi:10.3390/nu14051049)
Supplement: Supplementary file 1 [file nutrients-14-01049-s001.zip › Thai Clinical Trials Registry.pdf]

TCTR ID : TCTR20220209009

OTHER ID :

Overall Recruitment Status : Completed **(Has Results)**

Retrospective registration
This protocol was registered after enrollment of the first participant.

Tracking Information

First Submitted Date :	08 February 2022
First Posted Date :	09 February 2022
Last Update Posted Date :	09 February 2022

Title

Public Title :	Gummy Lactobacillus plantarum Dad-13 modulates gut microbiota in moderate undernutrition infants
Acronym :	No Data
Scientific Title :	Gut Microbiota Modulation of Moderate Undernutrition in In-fants through Gummy Lactobacillus plantarum Dad-13 Consumption: A Randomized Double-Blind Controlled Trial
Sponsor ID/ IRB ID/ EC ID :	3189/UN1.DITLIT/DITLIT/PT/2020
Registration Site :	Thai Clinical Trials Registry
URL :	https://www.thaiclinicaltrials.org/show/TCTR20220209009
Secondary ID :	Registry Identifier; Registry : https://ina-registry.org/ INA-DC4CNNS

Ethics Review

1. Board Approval :	Submitted, approved
Approval Number :	KE/FK/1303/EC/2019
Date of Approval :	06 November 2019
Board Name :	Medical and Health Research Ethics Committee
Board Affiliation :	Faculty of Medicine, Public Health, and Nursing, Universitas Gadjah Mada
Board Contact :	Business Phone : 274588688 Ext. +62
	Business Email : mhrec_fmugm@ugm.ac.id
	Business Address : Jl. Farmako, Sekip Utara, Yogyakarta 55128

Sponsor

Source(s) of Monetary or Material Supports :	Ministry of Research and Technology/National Research and Innovation Agency of Republic Indonesia (RISTEK-BRIN)
Study Primary Sponsor :	Ministry of Research and Technology/National Research and Innovation Agency of Republic Indonesia (RISTEK-BRIN)
Responsible Party :	Name/Official Title : Prof. Dr. Ir. Endang S Rahayu, MS
	Organization : Centre for Food and Nutrition Studies
	Phone : 274589242 Ext. +62
	Email : cfns@ugm.ac.id
Study Secondary Sponsor :	No Study Secondary Sponsor

Protocol Synopsis

Protocol Synopsis :	This study is a Randomized Double-Blind Controlled Trial and was conducted to evaluate the efficacy of gummy Lactobacillus plantarum Dad-13 (108-9 CFU/3 g) to prevent the progression of severe undernutrition. This study involves two groups of moderate undernutrition infants, namely the probiotic and placebo groups, who are required to consume the product for 50 days. Before and after the research product intervention, the stool samples will be collected for gut microbiota analysis, short-chain fatty acid, and stool quality. Also will be measured for their anthropometry.

URL not available

Health Conditions

Health Condition(s) or Problem(s) Studied :	moderate undernutrition
Keywords :	moderate undernutrition L. plantarum Dad-13 gut microbiota

Eligibility

This document was created on 10 February 2022 at 01:07 (GMT+7).

Inclusion Criteria : 1. Z score cut off between -2 and -3 standard deviation
2. not consuming probiotics, prebiotics, or antibiotics a month before the study

Gender : Both

Age Limit : Minimum : 24 Months Maximum : 60 Months

Exclusion Criteria : 1. refuse to consume research product
2. consume other probiotic products
3. consume antibiotic and laxative

Accept Healthy Volunteers : No

Status

Overall Recruitment Status : Completed

Key Trial Dates

Study Start Date (First enrollment) : 21 January 2020	Indicate Type : Actual
Completion Date (Last subject, Last visit) : 23 March 2020	Indicate Type : Actual
Study Completion Date : 23 March 2020	Indicate Type : Actual

Design

Study Type : Interventional

Primary Purpose : Prevention

Study Phase : Phase 1

Intervention Model : Parallel

Number of Arms : 2

Masking : Masked Masked Role : Allocation concealment, Subject, Caregiver, Investigator, Outcome Assessor, Statistician

Allocation : Randomized

Control : Placebo

Study Endpoint Classification : Safety/Efficacy Study

Sample size

Planned sample size : 40

Actual sample size at study completion : 40

Intervantion Arm 1

Intervention name : probiotic

Intervention Type : Active Comparator

Intervention Classification : Dietary Supplement

Intervention Description : The subjects are asked to consume gummy containing L. plantarum Dad-13 (108-9 CFU/3 g) once a day. The main ingredients of gummy consist of bovine gelatin, sucrose, glucose, water, and skim milk containing L. plantarum Dad-13.

Intervantion Arm 2

Intervention name : placebo

Intervention Type : Placebo Comparator

Intervention Classification : Dietary Supplement

Intervention Description : The subjects are asked to consume gummy without L. plantarum Dad-13 once a day. The main ingredients of gummy consist of bovine gelatin, sucrose, glucose, water, and skim milk without L. plantarum Dad-13.

Outcome

Primary Outcome

1. Outcome Name : Anthropometry

Metric / Method of measurement : body weight and body height

Time point : before-after

2. Outcome Name : Gut microbiota composition

Metric / Method of measurement : gut microbiota taxonomy and absolute number of specific bacteria

Time point : before-after

Secondary Outcome

1. Outcome Name : Dietary intake
Metric / Method of measurement : macro and micro nutrient intake
Time point : during intervention period

2. Outcome Name : SCFA
Metric / Method of measurement : SCFA concentration (Total SCFA, acetic, propionic, and butyric acid)
Time point : before-after

3. Outcome Name : stool pH
Metric / Method of measurement : stool pH
Time point : before-after

Location

Section A : Central Contact

Central Contact	First Name : Endang	Middle Name : Sutriswati	Last Name : Rahayu
	Degree : Prof. Dr	Phone : 8122690013 Ext. : 62	Email : endangsrahayu@ugm.ac.id
Central Contact Backup	First Name : Maryatun	Middle Name :	Lastname : Maryatun
	Degree :	Phone : 85643736235 Ext. : 62	Email : maria_slimshady@yahoo.com

Section B Facility Information and Contact

1. Site Name : Centre for Food and Nutrition Studies

	City : Sleman	State/Province : Yogyakarta	Postal Code : 55281
	Country : Indonesia	Recruitment Status : Completed	
**Facility Contact**	First Name : Endang	Middle Name : Sutriswati	Last Name : Rahayu
	Degree : Prof. Dr	Phone : 8122690013 Ext. : 62	Email : endangsrahayu@ugm.ac.id
**Facility Contact Backup**	First Name : Maryatun	Middle Name :	Last Name : Maryatun
	Degree :	Phone : 85643736235 Ext. : 62	Email : maria_slimshady@yahoo.com
**Investigator Name**	First Name : Rafli	Middle Name : Zulfa	Last Name : Kamil
	Degree : Dr	Role : Site Sub-Investigator	

Section C : Contact for Public Queries (Responsible Person)

First Name : Endang	Middle Name : Sutriswati	Last Name : Rahayu
Degree : Prof. Dr	Phone : 8122690013 Ext. : 62	Email : endangsrahayu@ugm.ac.id

Postal Address : Jl. Teknika Utara Barek, Yogyakarta

State/Province : Yogyakarta | Postal Code : 55281

Country : Indonesia | Official Role : Study Principal Investigator

Organization Affiliation : Centre for Food and Nutrition Studies

Section D : Contact for Scientific Queries (Responsible Person)

First Name : Endang	Middle Name : Sutriswati	Last Name : Rahayu
Degree : Prof. Dr	Phone : 8122690013 Ext. : 62	Email : endangsrahayu@ugm.ac.id

Postal Address : Jl. Teknika Utara Barek, Yogyakarta

State/Province : Yogyakarta | Postal Code : 55281

Country : Indonesia | Official Role : Study Principal Investigator

Organization Affiliation : Centre for Food and Nutrition Studies

---

Summary Results

Date of posting of results summaries : 08 February 2022
Date of first journal publication of results : Not yet published
Baseline Characteristics : The undernourished infants were prescreened according to their location, age, and the presence of a congenital disease. The infant's parents or guardians who passed the pre-screening were socialized the research background. The parents or guardians who agreed to participate in the study signed the informed consent and assent forms for further screening. The inclusion criteria were having the Z score cut-off between -2 and -3 standard deviation and not consuming probiotics, prebiotics, or antibiotics a month before the study. 1. (Average) Age probiotic group: 37.93 months, and placebo group: 37.80 months 2. (Average) Bodyweight probiotic group: 10.84 kg, and placebo group: 11.20 kg 3. (Average) Bodyheight probiotic

group: 87.06 cm, and placebo group: 88.88 cm Both groups were categorized as moderate undernutrition

Participant Flow : Forty infants passed the screening and were allocated for this study (20 infants for each group). However, only 15 subjects in each group finished the study. Five and four subjects in the placebo and probiotic groups resigned before the study began. One subject in the probiotic group did not collect the first stool sample.

Adverse events : No data

Outcome Measures : Primary outcomes: 1. There was a better improvement of anthropometry and nutritional status in the probiotic group, compared to the placebo group, even though the delta was not significantly different between groups. 2. L. plantarum Dad-13 modulated the butyric acid-producing bacteria to increase and inhibit the growth of Enterobacteriaceae. The number of L. plantarum was increased after the probiotic intervention. However, L. plantarum Dad-13 was not able to change the alpha and beta diversity. Therefore, L. plantarum Dad-13 has been proven to promote the growth of beneficial bacteria. Secondary outcomes: 1. There were no significant changes in macronutrient and fibre intake in both groups. 2. A notable difference was observed in the probiotic group, mainly the elevation of total SCFA, propionic, and butyric acid concentration after 50 days of intervention. In addition, a significant reduction of butyric acid was observed in the placebo group. However, the changes in SCFA concentration in both groups did not alter the stool pH significantly.

Brief Summary of Results : Delta Weight (kg): Probiotic: 0.59; Placebo: 0.39. Delta height (cm): Probiotic: 1.29; placebo: 1.29. Bacteria (Log 10 cells/g): Probiotic increment L. plantarum (pre: 4.85; post: 5.53), decrement Enterobacteriaceae (pre: 6.27; post: 5.80). Placebo (decrement of Bifidobacterium (pre: 6.24; post: 6.07). SCFA (mmol/g): Probiotic increment total SCFA, (pre: 23.55; post: 33.78), propionic (pre: 4.43; post: 6.89), and butyric (pre: 2.62; post: 4.67).

Deidentified Individual Participant-level Data Sharing

Plan to share IPD : No

Reason : Subject's privacy concern

Publication from this study

MEDLINE Identifier : No Data

URL link to full text publication : No Data
